# Hospital acquired pneumonia with high-risk bacteria is associated with increased pulmonary matrix metalloproteinase activity

**DOI:** 10.1186/1471-2466-8-12

**Published:** 2008-08-12

**Authors:** Bernhard Schaaf, Cornelia Liebau, Volkhard Kurowski, Daniel Droemann, Klaus Dalhoff

**Affiliations:** 1 Medical Clinic III, University of Lübeck, Lübeck, Germany; 2 Medical Clinic II, University of Lübeck, Lübeck, Germany

## Abstract

**Background:**

Neutrophil products like matrix metalloproteinases (MMP), involved in bacterial defence mechanisms, possibly induce lung damage and are elevated locally during hospital- acquired pneumonia (HAP). In HAP the virulence of bacterial species is known to be different. The aim of this study was to investigate the influence of high-risk bacteria like *S. aureus *and pseudomonas species on pulmonary MMPconcentration in human pneumonia.

**Methods:**

In 37 patients with HAP and 16 controls, MMP-8, MMP-9 and tissue inhibitors of MMP (TIMP) were analysed by ELISA and MMP-9 activity using zymography in bronchoalveolar lavage (BAL).

**Results:**

MMP-9 activity in mini-BAL was increased in HAP patients versus controls (149 ± 41 vs. 34 ± 11, p < 0.0001). In subgroup analysis, the highest MMP concentrations and activity were seen in patients with high-risk bacteria: patients with high-risk bacteria MMP-9 1168 ± 266 vs. patients with low-risk bacteria 224 ± 119 ng/ml p < 0.0001, MMP-9 gelatinolytic activity 325 ± 106 vs. 67 ± 14, p < 0.0002. In addition, the MMP-8 and MMP-9 concentration was associated with the state of ventilation and systemic inflammatory marker like CRP.

**Conclusion:**

Pulmonary MMP concentrations and MMP activity are elevated in patients with HAP. This effect is most pronounced in patients with high-risk bacteria. Artificial ventilation may play an additional role in protease activation.

## Background

Hospital-acquired pneumonia (HAP) is associated with high mortality rates of up to 30% in intensive care unit-related pneumonia [1], most prominent in ventilated patients [2]. Innate defense mechanism activating phagocytes locally in the lung play an important role in the elimination of bacteria, but overactivation might also be harmful to the host. Clinically, infections with *P. aeruginosa *and *S. aureus *are associated with the most severe HAP[1,3,4]. Besides bacterial virulence factors, the induction of the innate immunity might differ between different bacterial species.

An essential component of host defence against bacterial infection are polymorphonuclear neutrophils (PMN). In response to an inflammatory stimulus, PMN migrate into the alveolar compartment as primary effector cells to kill and phagocyte microorganisms. PMN are known to contain matrix metalloproteinases (MMP) [5]. MMP are a family of zinc- and calcium-dependent endopeptidases with 28 members to date that are subclassified into six groups. MMP-8 (neutrophil Collagenase) and MMP-9 (Gelatinase 2) are synthesized and stored in PMN [6]. During infection, antigen contact induces PMN activation and MMP release [7]. Elevated blood and bronchoalveolar lavage (BAL) levels of different MMP have been found in community and hospital-acquired pneumonia (8;9). MMP are thought to induce bacterial clearance possibly via induction of proinflammatory cytokines, since MMP knockout mice have a higher bacterial load and higher mortality after experimental infection [10]. Besides antimicrobial activity, free proteolytic activity of MMP might cause local tissue damage via degradation of different components of the extracellular matrix [11]. The possibility of local pulmonary damage is reduced via inhibitors of MMP, most importantly tissue inhibitors of MMP (TIMP) [6,12].

Apart from bacterial infection, mechanical ventilation might induce pulmonary inflammation. It is well-known that biotrauma associated with mechanical ventilation causes PMN recruitment [13]. MMP release and activation induced by cytokine release (IL-6, IL-8, TNF-alpha) are thought to be involved in lung damage in this setting [14].

Since both the type of bacterial infection and biotrauma due to invasive ventilation might influence the pulmonary release and activation of MMP, we asked the following questions:

1. Are infections with high-risk bacteria (*P. aeruginosa *and *S. aureus*) associated with a more pronounced pulmonary MMP release and activation than low-risk bacteria?

2. Is invasive ventilation associated with pulmonary MMP release and activation?

## Methods

### Study group

Thirty-seven patients with hospital-acquired pneumonia (HAP) were studied. Sixteen persons who underwent elective cardiac surgery were studied during ventilation (ventilation < 12 hours) as controls (controls published before [8], HAP patients not published before). The study protocol was approved of by the local ethics committee and informed written consent was obtained from all patients or close relatives.

### Definition of hospital-acquired pneumonia (HAP)

HAP was defined, according to ATS criteria adapted by Kollef et al. [4], as hospitalisation for > 48 hours, a new and persistent infiltrate (radiographically present for > 48 hours), PLUS at least two of the following criteria: [1] core temperature > 38.5 or < 36°C, [2] blood leukocytes > 10/μl or < 4/μl or [3] purulent tracheal secretions [4,15].

Only patients with a positive bacterial culture in mini-bronchoalveolar lavage [≥ 10^3 ^CFU/ml (colony forming units)] were included in the study.

Exclusion criteria were: age <= 18 years, blood leukocytes <= 1/μl, malignant hematologic disease, negative bacterial culture in mini-BAL.

### Pneumonia severity

The clinical severity of HAP was classified using the modified clinical pulmonary infection score (CPIS) described by Pugin [16]. In addition mortality, oxygenation index (arterial pO2/inspiratory O2 fraction: PaO2/FiO2) need of artificial ventilation and inflammatory markers (CRP, white blood count, temperature) were investigated.

### Mini-bronchoalveolar lavage

Mini-bronchoalveolar Lavage (Mini-BAL) was either performed during bronchoscopy in non-ventilated HAP-patients (n = 22) or via suction catheter in ventilated HAP-patients (n = 15) and ventilated controls (n = 16) [17]. The recovery was the same in both procedures.

#### Bronchoscopy

Bronchoscopy in non-ventilated patients was carried out under local anaesthesia with 2% lidocaine – after premedication with 2.5–7 mg midazolam – using a fibre-optic bronchoscope [18]. 5 ml 0.15 mol/l NaCl was instilled at the site of infection and immediately aspirated (recovered volume 3–4 ml).

#### Suction catheter

In ventilated patients 5 ml 0.15 mol/l NaCl was instilled over a sterile suction catheter below the carina and immediately aspirated (recovered volume 3–4 ml).

One ml of the recovered volume was used for bacterial culture, the other part (2–3 ml) was diluted with 20 ml of phosphate buffered saline (PBS), then homogenized and centrifuged (400 g for 10 minutes) to gain cell pellet and supernatant. The Mini-BAL supernatant was stored at -70°C in aliquots. The cell pellet was resuspended in Roswell Park Memorial Institute (RPMI) Medium. The cell count was carried out on a hemocytometer. Polymorphonuclear Neutrophil Granulocytes (PMN) were determined in a Wright-Giemsa stained cytocentrifuge smear, and a PMN vitality test was performed with Trypan Blue. The sample was then diluted to a concentration of 10^6 ^cells/ml.

### ELISA for matrix metalloproteinases (MMP) and tissue inhibitor of MMP (TIMP)

MMP-8, MMP-9 and TIMP-1 concentrations in Mini-BAL supernatant were determined by specific ELISA (Biotrak ELISA, Amersham Biosciences, Germany) following the manufacturer's instructions. Each sample was assayed in duplicate and the values used for calculations were all within the linear proportion of the standard curve.

### Gelatin zymography for matrix metalloproteinase activity

**Figure 1:**
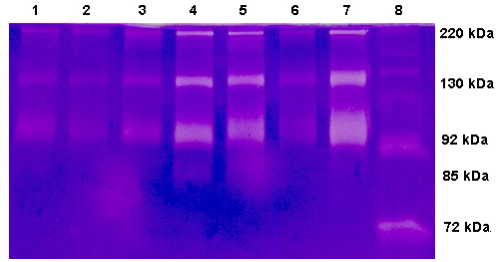
**Gelatine zymography of mini-BAL from representative patients with hospital-acquired pneumonia (Lane 1–7).** MMP standard (lane 8). Gelatinolytic bands of pro-MMP-9 (92 kD), pro-MMP-9-lipocalin-complex (130 kD) and homodimeric MMP-9 (220 kD) are clearly visible.

Sodiumdodecylsulfate gels containing 0.1% gelatine were used to identify gelatinolytic activity in Mini-BAL or plasma as described previously by Leber and Balkwill ([19], figure 1). MMP standard (MMP-2 and MMP-9) was loaded on each gel to identify the gelatinolytic enzymes. To quantify the gelatinolytic activity densitometrically, the bands were analyzed with E.A.S.Y.Win32 imaging software (Herolab, Germany). The bands were characterised in per-cent of the standard MMP-9 bands.

### Statistical analyses

Statistical analyses were carried out with Statistica for Windows, Statistica^® ^5.0 (Statsoft GmbH, Germany). Nonparametric tests such as Mann-Whitney U-test were used. Correlations were determined by the Spearman ranking test. Fisher's exact test (two tailed) was used for association of discontinuous variables. A p value of less than 0.05 was considered significant. The data are given as mean and standard error of the mean.

## Results

### Patients and controls

Demographic data of HAP patients are shown in table 1. Reason for hospital admission was surgery in 73% and cardiovascular insufficiency in 26%. As comorbidity, 21%t of the patients had chronic lung disease, 21% chronic heart disease, 3% diabetes mellitus, 8% chronic gastrointestinal disease and 24% solid neoplasia. Twenty-three percent had no comorbidity.

**Table 1:** Demographic data (Mean ± SEM) of 37 patients with HAP, including 17 patients with high-risk bacteria and 20 patients with low-risk bacteria.

	**all HAP patients (n = 37)**	**high-risk bacteria (n = 17)**	**low-risk bacteria (n = 20)**	*p-value
**number (n)**	37	17	20	
**age (years)**	59.8 (± 2.6)	59,3 (± 4,2)*	60,3 (± 3,5)*	*0.3
**male (%)**	68	70.6*	40*	*0.7
				
**WBC (/nl)**	12.3 (± 1.0)	13,0 (± 1,5)*	11,5 (± 1,5)*	*0.20
**temperature (°C)**	37.3 (± 0.18)	37,5 (± 0,2)*	37,0 (± 0,3)*	*0.45
**CRP (ng/ml)**	154.9 (± 29.5)	152,6 (± 28,9)*	157,9 (± 58,9)*	*0.51
				
ventilated n (%)*	15 (41%)	10 (59)*	5 (25) *	*0.05
**oxygenation ratio**	266.8 (± 21.5)	244,5 (± 33,9)*	284,6 (± 27,9)*	*0.4
				
**CPIS**	6 (± 0.35)	6,2 (± 0,4)*	5,0 (± 0,4)*	*0.24
**PMN in BAL (%)**	84,4 (± 3,2)	83,6 (± 4,1)*	87,3(± 1,6)*	*0,71
**mortality (%)**	8.1	5.9*	10*	*1.0

*p- value between patients with high-risk and low-risk bacteria.

### Bacterial culture in mini-BAL

Bacterial culture data from Mini-Bal are shown in table 2. From 37 patients with HAP, 17 patients had high-risk bacteria (*S. aureus*, *P. aeruginosa or S. maltophilia) *and 20 patients low-risk bacteria (Table 2). Mini-BAL from the control patients remained sterile (control group).

**Table 2:** Recovered bacteria in Mini-BAL from 37 HAP patients (≥ 10^3 ^CFU/ml).

**Bacteria in Mini-BAL**	**Number of patients**
High-risk bacteria	
• *S. aureus*	9
• *P. aeruginosa*	7
• *S. maltophilia*	1
Low-risk bacteria	
• *Klebsiella pneumoniae*	4
• *E. coli*	3
• *S. marcescens*	2
• *S. pneumoniae*	2
• β-hemolytic *Streptococci*	2
• *Citrobacter freundii*	1
• *Enterobacter cloacae*	1
• *Hafnia alvei*	1
• *P. mirabilis*	1
• *N. meningitidis*	1
• *Branhamella catarrhalis*	1
• Chlamydia pneumoniae*	1

* PCR

### MMP and TIMP in HAP versus control

When comparing HAP Patients with controls, MMP-8, MMP-9 concentrations were higher and TIMP-1 lower in Mini-BAL of HAP patients vs. controls without reaching significance (Table 3). The molar MMP-9/TIMP-1 ratio and the gelatinolytic activity of MMP-9 was increased in HAP patients compared to controls (MMP-9/TIMP-1 ratio 0.43 (± 0.12) vs. 0.04 (± 0.02), p < 0.0001; MMP-9 activity 149 (± 41) vs. 34 (± 11), p = 0.0001). The activated form of MMP-9 (85 kDa) was seen in 68% versus 0% of controls (p < 0.0001).

**Table 3:** MMP, TIMP, MMP-9 activity and molar ratio in patients with HAP and control patients.

	**Mean ± SEM**	**p- value**	
**MMP-8 **(ng/ml)			
Control group (n = 18)	24 (± 7)	HAP vs. control	0.346
All HAP patients (n = 37)	435 (± 131)		
HAP subgroups			
• Bacteria		high vs. low risk	0.002
○ high-risk bacteria (n = 17)	750 (± 245)	high vs. control	0.0007
○ low-risk bacteria (n = 20)	167 (± 93)	low vs. control	0.198
• invasive ventilation			
○ ventilated (n = 15)	891 (± 266)	vent. vs. not vent.	<0.0001
○ not ventilated (n = 22)	124 (± 77)		
			
**MMP-9 **(ng/ml)			
Control group (n = 18)	50 (± 17)*	HAP vs. control	0.059
All HAP patients (n = 37)	657 (± 157)*		
HAP subgroups			
• Bacteria		high vs. low risk	<0.0001
○ high-risk bacteria (n = 17)	1168 (± 266)**	high vs. control	<0.0001
○ low-risk bacteria (n = 20)	224 (± 119)**	low vs. control	0.497
• invasive ventilation			
○ ventilated (n = 15)	1309 (± 266)***	vent. vs. not vent.	<0.0001
○ not ventilated (n = 22)	213 (± 131)***		
			
**MMP-9 gelatinolytic activity **(%)			
Control group (n = 18)	34 (± 11)*	HAP vs. control	0.0001
All HAP patients (n = 37)	149 (± 41)*		
HAP subgroups			
• Bacteria		high vs. low risk	0.0001
○ high-risk bacteria (n = 17)	325 (± 106)**	high vs. control	<0.0001
○ low-risk bacteria (n = 20)	67 (± 14)**	low vs. control	0.007
• invasive ventilation			
○ ventilated (n = 15)	197 (± 40)***	vent. vs. not vent.	0.0024
○ not ventilated (n = 22)	118 (± 63)***		
			
**TIMP-1 **(ng/ml)			
Control group (n = 18)	410 (± 80)*	HAP vs. control	0.275
All HAP patients (n = 37)	648 (± 164)*		
HAP subgroups			
• Bacteria		high vs. low risk	0.0039
○ high-risk bacteria (n = 17)	983 (± 285)**	high vs. control	0.290
○ low-risk bacteria (n = 20)	364 (± 165)**	low vs. control	0.005
• invasive ventilation			
○ ventilated (n = 15)	1309 (± 261)***	vent. vs. not vent.	<0.0001
○ not ventilated (n = 22)	132 (± 40)***		
			
**MMP-9/TIMP-1 molar ratio**			
Control group (n = 18)	0.04 (± 0.02)*	HAP vs. control	<0.0001
All HAP patients (n = 37)	0.43 (± 0.12)*		
HAP subgroups			
• Bacteria		high vs. low risk	0.0002
○ high-risk bacteria (n = 17)	0.66 (± 0.17)**	high vs. control	<0.0001
○ low-risk bacteria (n = 20)	0.24 (± 0.15)**	low vs. control	0.0028
• invasive ventilation			
○ ventilated (n = 15)	0.66 (± 0.23)***	vent. vs. not vent.	0.0046
○ not ventilated (n = 22)	0.28 (± 0.12)***		

Subgroups according to high/low-risk bacteria and artificial ventilation.

The results for MMP-9 in mini-BAL correlated significantly with the densitometric evaluation of MMP-9 in Zymography (r = 0.79; p < 0.001).

### MMP and TIMP according to bacteria

**Figure 2:**
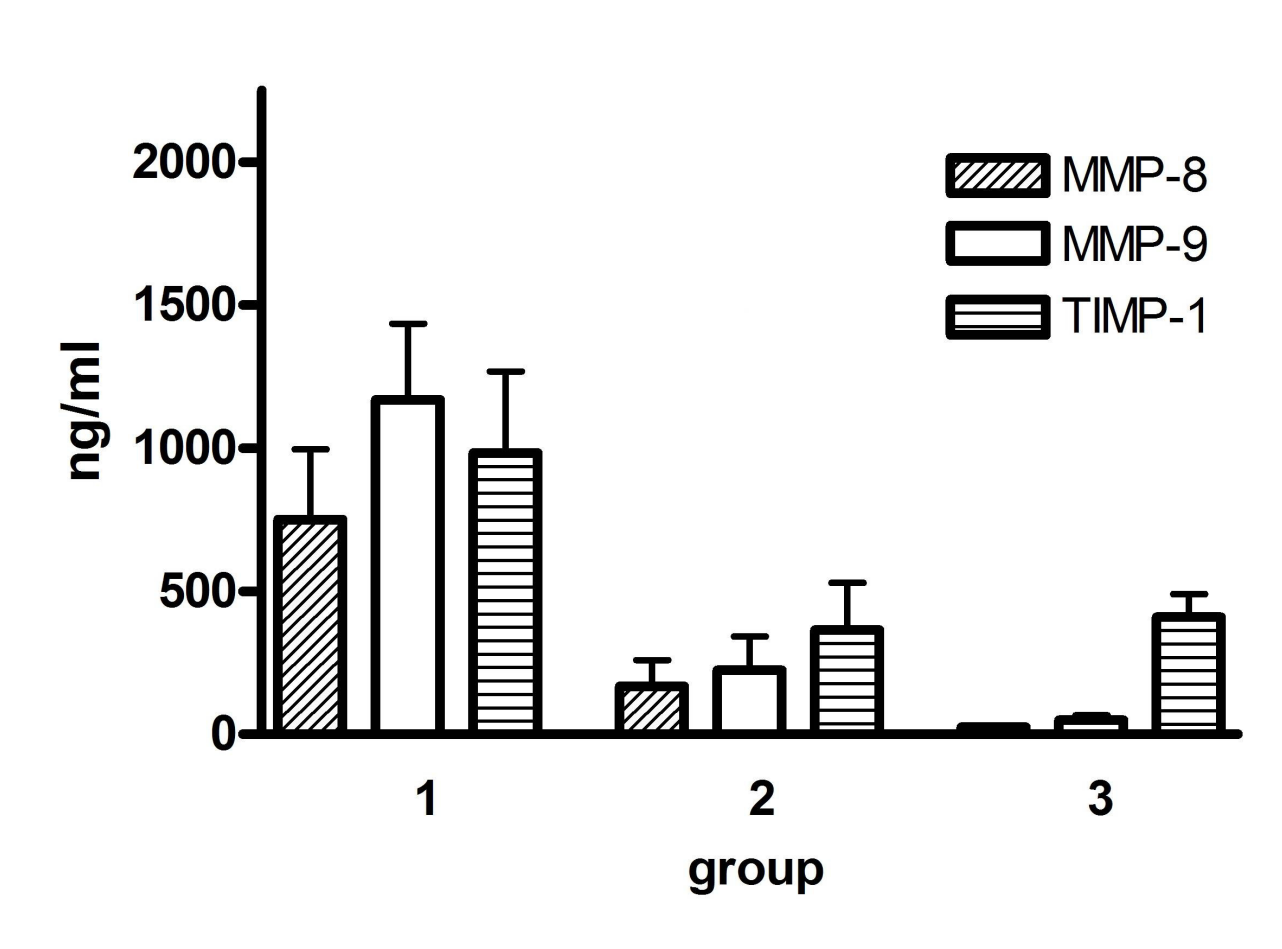
**MMP-8, MMP-9 and TIMP-1 concentration in mini-BAL from HAP patients and controls.** Group 1 = HAP patients with high risk bacteria; group 2 = HAP patients with low-risk bacteria; group 3 = control patients (Mean and SEM).

In Patients with high-risk bacteria (*S. aureus*, *P. aeruginosa or S. maltophilia *infection), MMP-8-, MMP-9- and TIMP-1-concentrations, the MMP-9 gelatinolytic activity and the MMP-9/TIMP-1 molar ratio in mini-BAL were higher than in patients with low-risk bacteria (MMP-8 p = 0.002; MMP-9 p < 0.0001; TIMP-1 p = 0.0039; MMP-9 gelatinolytic activity p = 0.0002, molar ratio p = 0.0002) and control patients (Table 3, figure 2). In addition, patients with high-risk bacteria had more often (16 out of 17) the activated form of MMP-9 (85 kDa) than patients with low-risk bacteria (9 out of 20, p < 0.005) and controls (0 out of 16, p < 0.0001)

Interestingly, in patients with low-risk bacteria MMP-8, MMP-9 concentrations in mini-BAL were indifferent to controls (Table 3), but TIMP-1 concentrations were lower (p = 0.005) associated with higher MMP-9 activity (p = 0.007) and MMP-9/TIMP-1 ratio (p = 0.003) than in controls (Table 3).

The quantitative pathogen count (CFU/ml) in Mini-BAL did not correlate with MMP- and TIMP-levels in Mini-BAL and blood (data not shown). Plasma levels did not differ significantly between patients with high-risk and low-risk bacteria (data not shown).

### MMP and TIMP according to artificial ventilation

Ventilated patients (n = 15, all invasive ventilation with pressure controlled/BIPAP modus) had significantly higher MMP-8, MMP-9 and TIMP-1 concentrations, gelatinolytic MMP-9 activity and MMP-9/TIMP-1 molar ratio in mini-BAL than non-ventilated patients (n = 22) (MMP-8 p < 0.0001; MMP-9 p < 0.0001; TIMP-1 p < 0.0002; MMP-9 activity p < 0.005, molar ratio p < 0.006).

In zymography, the activated form of MMP-9 (85 kDa band) was seen in 93% of ventilated patients vs. 50% of non ventilated patients (p < 0.005).

**Figure 3:**
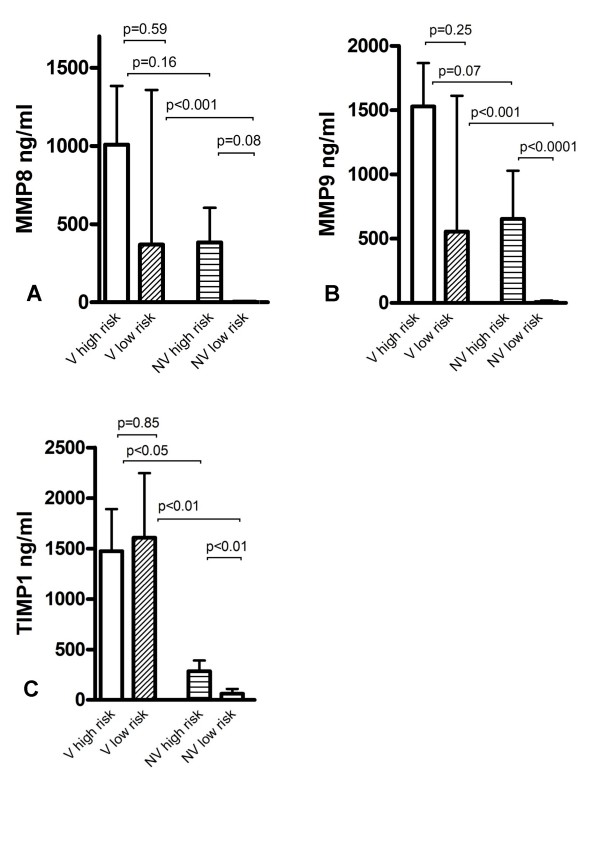
**MMP-8 (A), MMP-9 (B) and TIMP-1 (C) concentration in mini-BAL from patients with high-risk and low-risk bacteria in association with artificial ventilation.** V = ventilated, NV = not ventilated.

Ventilation time (≤ 7 days vs. >7 days) did not have an impact on MMP-levels in Mini-BAL (data not shown). Interestingly, invasive ventilation and high-risk bacteria seem to be additive for the increase of the MMP concentration in BAL (figure 3).

MMP plasma levels did not differ significantly between ventilated and non-ventilated patients (data not shown). TIMP-1 and the MMP-9/TIMP-1 molar ratio in plasma was higher in ventilated patients (TIMP-1 p = 0.014, molar ratio p = 0.046)

### MMP and TIMP in correlation to clinical and laboratory parameters

#### Pneumonia severity

In HAP patients no correlation was found between MMP-levels in Mini-BAL and the oxygenation index, the CPIS score or the PMN Mini-BAL percentage (data not shown).

#### Systemic inflammation (figure 3)

**Figure 4:**
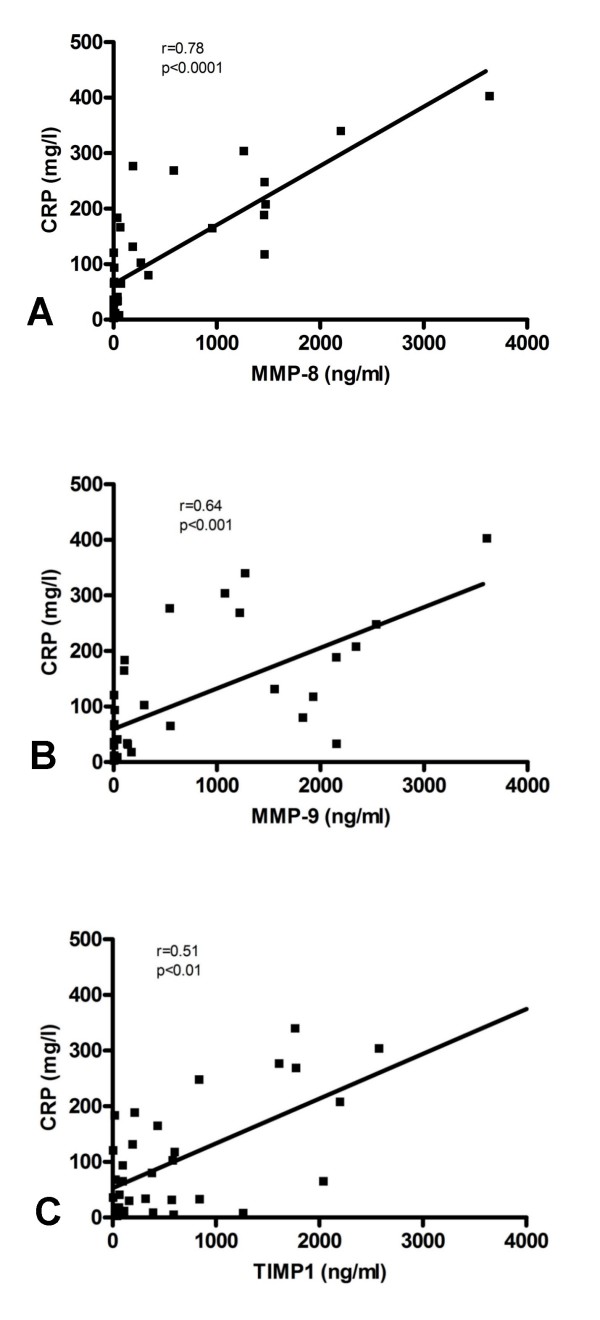
Correlation of MMP-8 (A), MMP-9 (B) and TIMP-1 (C) concentration in mini-BAL with serum CRP in patients with hospital-acquired pneumonia.

In HAP patients all Mini-BAL-concentrations correlated with serum-CRP (MMP-8: r = .78; p < .0001; MMP-9: r = .64; p < .0001; MMP-9 activity r = .69; p < 0.001; TIMP-1: r = .51; p = .002, MMP-9/TIMP-1 molar ratio r = .50; p = 0.002, figure 4), most Mini-BAL-concentrations correlated with body temperature (MMP-8: r = .52; p = .004; MMP-9: r = .35; p = 0.065, n.s.; MMP-9 activity r = .55; p = 0.014; TIMP-1: r = .48; p = .009; MMP-9/TIMP-1 molar ratio r = .08; p = 0.69, n.s.) and most Mini-BAL-concentrations correlated with serum leukocytes (MMP-8: r = .42; p = .008; MMP-9: r = .34; p = .034; MMP-9 activity r = .60; p = 0.0005; TIMP-1: r = .34; p = 0.04, MMP-9/TIMP-1 molar ratio r = .26; p = 0.11, n.s.).

## Discussion

The main finding of our study is that nosocomial pulmonary infection with high-risk pathogens (*S. aureus*, *P. aeruginosa or S. maltophilia) *is associated with higher concentration and activation of metalloproteases locally in the lung, than nosocomial infection with other bacteria. In addition artificial ventilation was associated with additional increased MMP concentration and activation.

The role of the TIMP-MMP axis during inflammatory responses seems to be complex, data are limited.

Bacterial infection of the lower respiratory tract induces a proinflammatory milieu with the activation of MMP and inactivation of TIMP via several mechanisms. Attracted to the lung by IL-8 and leukrotrien B4 PMN secrete MMP-8 and MMP-9. MMP are activated by bacterial MMP [20], plasmin [21] or other neutrophil products such as myeloperoxidase and neutrophil elastase (NE). In addition NE inhibits TIMP-1 [22].

In community-acquired pneumonia (CAP) Yang et al. found an excessive increase of MMP-9 activity and MMP-9 levels in plasma [9] but the local pulmonary TIMP-MMP balance was not investigated. Hartog et al. found increased activated pulmonary MMP in hospital-acquired pneumonia (HAP) with some evidence of increased MMP levels in patients with proven bacterial infection [8]. In line with these studies, we found in our study with HAP patients higher MMP-8 and MMP-9 BAL concentrations associated with significantly increased MMP-9 activity. A lot of data shows that, in chronic pulmonary diseases, an imbalance of proteases and inhibitors can induce consecutive degradation of the extracellular matrix of the lung [23]. Increased MMP secretion in association with cigarette smoke is discussed as a factor in COPD development and progression of COPD [24]. Segura-Valdez et al. have found increased levels of MMP-8 and MMP-9 in Mini-BAL and lung biopsies of COPD patients [25]. Zheng et al did show in bronchiectasis [26] neutrophil inflammation associated with MMP-8 and MMP-9 concentration. Sagel et al. have shown that cystic fibrosis (CF) patients show significantly elevated MMP levels with increased MMP-9/Timp-1 molar ratios [27]. Taghavi and coworkers have described increased MMP-9 and MMP-8 levels in bronchiolitis obliterans from lung transplant patients [28]. In addition, various lung injury models show that MMPs are strongly related to the pathogenesis of lung injury [29-31]. The role of increased MMP-8 and MMP-9 concentrations and activity in bacterial infection of the lung is, in contrast to chronic disease, under debate. Besides tissue destructive effects, increased MMP activation might be involved in host defence mechanism inducing bacterial eradication [32] and might have a protective effect against pulmonary fibrosis following inflammation [33]. In animal models MMP-9 deficiency is associated with impaired host defence against abdominal sepsis, with reduced bacterial clearance [10]. MMP-9 is known to be a major factor in neutrophil migration across basement membranes [34]. MMP-9 -/- mice displayed diminished recruitment of leucocytes to the site of infection [10]. On the other hand experimental pulmonary infections with *Francisella tularensis *in MMP-9 wildtype mice did show an increased neutrophil inflammation of the lung accompanied by increased bacterial burden and mortality compared to MMP-9 -/- animals [35].

Since pathogens interact with the TIMP-MMP axis, induction of the MMP concentration and activity might be related to different species. Oggioni et al. have found a pneumococcal zinc metalloproteinase to cleave and thus to activate MMP-9 in a murine model of pneumonia and meningitis. In certain mutants this leads to a more severe course of disease than in other isolates of pneumococci [20]. This indicates that different bacterial strains and species have a different impact on MMP secretion and activation. Indeed, in the present study, in patients with high-risk pathogens (*S. aureus*, *P. aeruginosa or S. maltophilia) *we found significantly higher MMP-8 and MMP-9 BAL concentration locally in the lung associated with increased MMP-9/Timp-1 molar ratio and MMP-9-activity compared to patients with low-risk bacteria and to controls (Table 3). High-risk pathogens seem to induce a strong neutrophil MMP release. In contrast, patients with low-risk bacteria have, compared to controls, only an insignificant increase of MMP. Due to low inhibitor concentrations, these patients still have active MMP-9 (Table 3). Low MMP increment and activation in HAP-patients with low-risk bacteria might be explained by less virulence of these bacteria. In addition, a positive bacterial culture in patients with a clinical diagnose of HAP according to the ATS criteria [4] is not 100% specific to detect HAP. Compared to post-mortal histopathology, the specificity of the ATS criteria to define HAP is only 70 to 80% [36]. Some of the HAP patients with low-risk bacteria (diagnosed by ATS criteria) and low MMP activation might not have significant pneumonia when histopathologic examination is performed. Clinicians should be aware that detecting bacteria does not always distinguish between airway colonization and invasive infection.

Interestingly we found in HAP patients a close correlation between pulmonary MMP levels and laboratory parameters of systemic inflammation. Increased pulmonary MMP was associated with higher serum-CRP, higher white blood count and higher body temperature (Figure 2). MMP activation in HAP might be necessary for bacterial clearance, but unresolved infection might trigger ongoing MMP activation and tissue destruction[26,27]. MMP concentration and activation seems to be a marker for the severity of inflammation and might be, in case of very low concentration, a diagnostic tool.

In addition to bacterial infection, ventilation associated lung trauma and inflammation is of great interest. Foda et al. discussed the role of MMP in ventilator-induced lung injury [14]. They suggested high-volume ventilation as a leading cause of lung injury. This is due to release of TNF-alpha and interleukines and thus upregulation of MMP. Halbertsma states that the biotrauma associated with mechanical ventilation causes cytokine release (IL-6, IL-8, TNF-alpha) with consecutive PMN recruitment to the lung [13]. In spite of the fact that all ventilated patients have been ventilated with modern pressure control ventilation, we found significantly higher MMP and Timp-1 levels in BAL from ventilated patients compared to non-ventilated patients. Since 2/3 of the ventilated patients were infected with high-risk bacteria, these data are difficult to evaluate. When looking at subgroups, both factors, ventilation and high-risk bacteria, seem to have an additive effect on MMP release and activation (figure 2). HAP-Patients with high-risk bacteria and ventilation have the highest values. In line with this, it has been shown in animal models that both stretch during ventilation and stimulation with bacterial antigen, activate nuclear factor B, followed by cytokine release [37]. Mechanical stretch of human lung endothelium is associated with MMP release [38]. Other authors have shown an upregulation of MMP during ventilator associated lung injury in a rat model. In this setting, inhibition of MMP activity reduced lung injury [14].

## Conclusion

In conclusion high amounts of active MMP are found in BAL from patients with HAP, which is most prominent in patients with high-risk bacteria and patients with artificial ventilation. Bacteria and ventilation trauma seems to induce neutrophil inflammation and metalloprotease activation. The pulmonary MMP concentration is closely correlated to systemic signs of inflammation. The pathophysiologic role of this local MMP-inflammation is under debate.

## Competing interests

The authors declare that they have no competing interests.

## Authors' contributions

CL carried out the ELISA and zymography and was involved in the design of the study and drafting the manuscript. BS, KD, VK and DD conducted the clinical part of the study, were involved in the design and coordination of the study and drafting the manuscript. All authors read and approved the final manuscript.

## Pre-publication history

The pre-publication history for this paper can be accessed here:


